# A Bayesian optimization R package for multitrait parental selection

**DOI:** 10.1002/tpg2.20433

**Published:** 2024-02-22

**Authors:** Bartolo de J. Villar‐Hernández, Susanne Dreisigacker, Leo Crespo, Paulino Pérez‐Rodríguez, Sergio Pérez‐Elizalde, Fernando Toledo, José Crossa

**Affiliations:** ^1^ International Maize and Wheat Improvement Center (CIMMYT) Estado de México México; ^2^ Colegio de Postgraduados Montecillo Estado de México 56230 México

## Abstract

Selecting and mating parents in conventional phenotypic and genomic selection are crucial. Plant breeding programs aim to improve the economic value of crops, considering multiple traits simultaneously. When traits are negatively correlated and/or when there are missing records in some traits, selection becomes more complex. To address this problem, we propose a multitrait selection approach using the Multitrait Parental Selection (MPS) R package—an efficient tool for genetic improvement, precision breeding, and conservation genetics. The package employs Bayesian optimization algorithms and three loss functions (Kullback–Leibler, Energy Score, and Multivariate Asymmetric Loss) to identify parental candidates with desirable traits. The software's functionality includes three main functions—EvalMPS, FastMPS, and ApproxMPS—catering to different data availability scenarios. Through the presented application examples, the MPS R package proves effective in multitrait genomic selection, enabling breeders to make informed decisions and achieve strong performance across multiple traits.

## Core Ideas

- The Multitrait Parental Selection (MPS) R package aids plant and animal breeders in addressing multitrait parental selection in both phenotypic and genomic selection.
- MPS incorporates Bayesian optimization algorithms into genomic selection by employing various loss functions.
- The results generated by MPS can be utilized to identify the most promising parental candidates based on genetic gain and diversity.
- The MPS R package seamlessly integrates with the widely used Bayesian Generalized Linear Regression (BGLR) software.

**Abbreviations**
BDT: Bayesian Decision Theory
BGLR: Bayesian Generalized Linear Regression
BV: breeding value
EnergyS: Energy Score
GBLUP: Genomic Best Linear Unbiased Prediction
GS: genomic selection
KL: Kullback–Leibler
LF: loss function
MALF: Multivariate Asymmetric Loss Function
MCMC: Markov Chain Monte Carlo
MPS: Multitrait Parental Selection
M×E: Markers–Environment interaction model
NA: not available
PEL: posterior expected loss
RKHS: Reproducing Kernel Hilbert Space
SNP: single‐nucleotide polymorphism

## 1 INTRODUCTION

Developing software in genomic selection (GS) to face multitrait selection holds significant importance in advancing our understanding of complex traits and improving the efficiency of breeding programs in various fields such as agriculture, livestock, conservation genetics, and so forth. For example, plant breeders are interested in individuals that exhibit high breeding values (BVs) for traits such as grain yield, protein content, and baking quality in wheat breeding programs (Michel et al., 2019). Sometimes, one or more traits conflict with others, and selection becomes difficult because these traits act antagonistically (negatively correlated), as in the previous example. In such cases, selection becomes more challenging and can be more successfully addressed within a multitrait scheme using appropriate software. In favorable instances where the traits are positively correlated, multitrait selection can improve the overall performance and adaptability of crop varieties exploiting the genetic correlation of the traits of interest. This advantage should be considered when developing software.

However, multitrait selection goes beyond merely exploiting the genetic correlation of the traits of interest per se. Climate change also imposes new challenges for which plant breeders previously did not account, such as altered pluvial regimens, increased temperatures, and more frequent extreme weather events. Therefore, plant breeders need to develop crop varieties that can be better adapted to these changing conditions (Suter et al., 2021; Yue et al., 2022). With multitrait selection software, we can efficiently develop adaptable crop varieties that can withstand these challenges, ensuring sustainable agricultural practices and food security. It should be noted that multitrait selection is not only limited to genetic improvement in agriculture or animal science; along with GS, it also has important applications in fields such as forest preservation and restoration (Laverdière et al., 2022; Lenz et al., 2020) and biodiversity (Savage et al., 2007).

Advantages of multitrait selection also impact efficient resource utilization because breeders can optimize time, labor, land, and financial investments. Instead of improving one trait a time, multitrait selection can enable breeders to work on multiple targets simultaneously, accelerating the breeding process (Gevartosky et al., 2023; Lado et al., 2018; Torres et al., 2018). From a consumer's point of view and market demands, multitrait selection allows plant breeders to face an increasing demand for crop varieties with specific attributes, such as improved taste, nutritional value, or suitability for specific processing methods.

But what does multitrait selection mean? When breeders perform selection, they need to identify, among a candidate set of potential individuals, which ones should be parents of the next improved population. If selection operates in a single trait, we can select a fraction of candidate's individuals that rank high based on estimated BVs (selection by truncation). The selection response measures the genetic gain throughout successive selection cycles. In multitrait scenario, the criterion for selecting the best individuals in a population becomes more complex.

The traditional methodology to deal with the multitrait selection problem is the use of selection indices, which assign subjective economic weights to each trait (Smith, 1936). Moreover, there are alternatives, for example, optimizing a subset of parents using pedigree‐based co‐ancestry information and additive genetic values approach (Shepherd & Kinghorn, 1998), and algorithms that minimize inbreeding and co‐ancestry through quadratic optimization, which considers the context of co‐ancestry information and additive genetic values (Brisbane & Gibson, 1995; Wray & Goddard, 1994).

More recently, Akdemir and Sánchez (2016) introduced a genetic algorithm designed to optimize genomic mating strategies among parents in GS. They introduced what is called a risk function of a mating plan, which integrates both the expected mean and variance among progenies. By weighing the risk function of a given candidate breeding scheme against the expected inbreeding level for the plan, breeders can select a mating scheme that minimizes inbreeding for a given risk value.

The concept of selection by truncation, commonly applied in single‐trait selection, can be extended to the realm of multitrait selection. Through the incorporation of the Bayesian Decision Theory (BDT) approach, a discerning metric can be formulated to assess candidate individuals. The score or expected loss serves as a valuable criterion for ranking individuals. Superior candidates are identified as those exhibiting the least expected losses. Consequently, a subset of candidates characterized by the lowest expected losses is preferred as the progenitors for the subsequent generation, thereby contributing to the advancement of the breeding program. This approach demonstrates the efficacy of utilizing expected losses as a discriminative measure to strategically guide the selection of individuals for enhanced genomic improvement. Both approaches—truncated selection and optimizing a genetic mating system—can be considered as decision problems under uncertainty.

The methodology based on BDT was introduced by Villar‐Hernandez et al. (2018) as a formal methodology for selecting parents in the context of single‐ and multitrait GS in plant breeding. They introduced three univariate loss functions (LFs) (Kullback–Leibler [KL]; Continuous Ranked Probability Score [CRPS]; and Linear–Linear loss [LinLin]) and their corresponding multivariate generalizations (KL; Energy Score [EnergyS]; and the Multivariate Asymmetric Loss Function [MALF]). The authors derived these LFs and expressed them in terms of trait heritability. Results were obtained and compared with those using the standard selection method based on point predictions of BVs in a single‐trait scenario. Their findings suggested that in a single‐trait context, selecting based on LFs can lead to better performance in a long‐term breeding program compared to standard selection method in some cases. For the multitrait approach, our results demonstrate positive gains in the population mean for all considered traits, even when two traits were negatively correlated. While each multivariate LF performed well in terms of population means, there were no statistically significant differences in population variances.

Following the initial investigation, more recently Villar‐Hernández et al. (2021) conducted multitrait selection using real wheat datasets with four traits and employed the BDT framework and the concept of LFs to explore practical applications and address gaps in a previous study (Villar‐Hernández et al., 2018). The three LFs used for multitrait selection were KL, EnergyS, and MALF. Authors’ findings indicated that all LFs performed similarly in selecting the best individuals for maximizing genetic progress across all traits. However, in one dataset, EnergyS and MALF showed a slight advantage over KL, reporting greater gains in three out of four traits. Regarding genetic variance, the three LFs exhibited similar performance in both datasets. Although LFs occasionally selected lines with high or low/medium variance, the overall differences in variance compared to the whole population were insignificant. Selection using LFs has the potential to be effective in multitrait selection within GS, as it incorporates important genetic concepts such as heritability, response to selection, and trait correlations.

Creating software for the selection of parents in genomics provides a powerful tool for genetic improvement, precision breeding, conservation genetics, and resource optimization. It enhances the efficiency, accuracy, and speed of breeding programs, leading to improved outcomes in agriculture, livestock, and conservation efforts. The selection of parents using GS software is crucial for (1) identifying individuals with desirable traits and using them as parents, which can lead to enhanced crop yields, disease resistance, and overall productivity in agriculture; (2) speeding up the breeding process by providing rapid and accurate predictions of an individual's genetic merit; (3) maintaining genetic diversity and avoiding inbreeding, which can help conservationists identify individuals with high genetic diversity and prioritize them as potential parents for breeding programs; (4) enabling the integration and analysis of vast amounts of genetic data allowing researchers to combine genotypic and phenotypic information from various sources and analyze it in a systematic and efficient manner, enhancing our understanding of complex genetic traits, such as disease susceptibility or yield potential, which facilitates the identification of superior parental combinations; and (5) resource optimization: by selecting the most suitable parents based on genetic data, resources such as land, labor, and inputs can be utilized more efficiently—by minimizing the number of unsuccessful breeding attempts, GS software reduces costs associated with trial‐and‐error breeding approaches.

In this article, we present an R package software to facilitate the multitrait selection of parental candidates, primarily in GS in animal and plant breeding. The software uses a Bayesian optimization algorithm, utilizing three LFs for selecting parental candidates. Throughout the article, we will provide the reader with a comprehensive overview of the scope of our proposal by presenting some simple application examples. The theory of our approach can be found in previous publications of Villar‐Hernández et al. (2018, 2021). Our goal is to develop a package that can be integrated into the Bayesian Generalized Linear Regression (BGLR) software (Pérez‐Rodríguez & de los Campos, 2022), which fits many genomic prediction models and enjoys broad acceptance within the breeding community.

**Core Ideas**

- The Multitrait Parental Selection (MPS) R package aids plant and animal breeders in addressing multitrait parental selection in both phenotypic and genomic selection.
- MPS incorporates Bayesian optimization algorithms into genomic selection by employing various loss functions.
- The results generated by MPS can be utilized to identify the most promising parental candidates based on genetic gain and diversity.
- The MPS R package seamlessly integrates with the widely used Bayesian Generalized Linear Regression (BGLR) software.

## 2 MATERIALS AND METHODS

### 2.1 Installation of MPS package and its main functions

MPS (Multitrait Parental Selection) R Package is hosted on Github (https://github.com/bjesusvh/MPS) and can be installed by executing in the R console devtools::install_github(“bjesusvh/MPS”). After installation, we can load into R environment as usual: library(MPS). The main functions are EvalMPS, FastMPS, and ApproxMPS. Users can get help how to use the main functions typing, for example, help(FastMPS). While both EvalMPS and FastMPS receive the same inputs, FastMPS was designed to run in parallel to achieve faster computations. ApproxMPS receives different arguments because it was designed to integrate with pedigree (A), to interface with genomic relationship matrix (G), or for scenarios where there are dozens of traits available, making the evaluation of the expected loss via Markov Chain Monte Carlo (MCMC) slow and computationally expensive. In other words, FastMPS and EvalMPS should be used when all MCMC samples are available, while ApproxMPS relies on point estimates only. Figure S1 depicts a summarized workflow for using BDT within the MPS R package.

**Table:** 

FastMPS(Xcand, B0, B, R, target = NULL, method = "kl", direction = NULL)
EvalMPS(Xcand, B0, B, R, target = NULL, method = "kl", direction = NULL)
ApproxMPS(B0, yHat, R, target = NULL, method = "kl", direction = NULL)

Below is a brief overview of each of the required arguments in the aforementioned functions. In the results section, detailed explanations are presented.

- Xcand is a matrix of predictors of the candidates for selection; its dimensions are n×k, where n is the number of individuals in the candidate set and k is the number of predictors.
- In EvalMPS and FastMPS, B0 represent a matrix of dimension M×t for the intercept term in the Bayesian linear model previously fitted using the function Multitrait of the BGLR R package. M is the number of MCMC samples and t is the number of traits. In the case of ApproxMPS, B0 is a vector with the overall mean of each trait of length equal to number of traits, t.
- B corresponds to an array containing regression coefficients of dimension M×k×t of MCMC samples for EvalMPS and FastMPS.
- yHat is only used in ApproxMPS and corresponds to a matrix of posterior mean of each candidate of dimension n×t, where n is the number of individuals in the candidate set.
- R denotes a matrix of dimension M×(t×(t+1)/2) of MCMC samples of the variance–covariance components in the residual covariance matrix when using EvalMPS or FastMPS, while in ApproxMPS, it represents the square matrix of the residual covariance of dimension t×t.
- target is a real vector of length equal to the number of traits (t) reflecting the breeder's expectation after selection. The default is NULL and it is calculated internally.
- method indicates the LF to be used. This must be one of “kl” for KL, “energy” for EnergyS, and “malf” for Multivariate Asymmetric Loss. The default is “kl.”
- direction argument is a vector of length equal to the number of traits (t) reflecting the direction of improvement desired, 1 for increase goal and −1 for decreasing goal. The default is 1 (increase) for all traits.
- There are other minor functions in MPS package such as SimMatrix, AveSim, and AveDist discussed in the next section.

### 2.2 Theory of loss functions

The theory of LFs, used to enhance genetic gain in selection programs, was developed in our previous publications (Villar‐Hernández et al., 2018, 2021). The foundation of LF theory stems from the concept of selection by truncation in single traits and extends to address multitrait selection. Previous works introduced three LFs for multitrait selection: KL, EnergyS, and MALF.

The underlying idea is to calculate the expected distance (divergence) between the theoretical multivariate distribution when selection by truncation ($F_{Y_{p}}$) occurs and the multivariate distribution of a candidate line for selection ($F_{Y_{c}}$). The goal is to identify those candidate lines to be parents for which this expected distance is minimized.

The posterior expected loss (PEL) or distance of each line is formally obtained in the next equation, where p(θ|y,X) corresponds to joint posterior distributions of the model's parameters coming from whatever Bayesian multitrait linear models (e.g., Spike–Slab, Gaussian, etc.), and $f(y_{c}|\theta ,{x^{\prime }}_{c})$ denotes the sampling model (multivariate normal) with $x_{c}^{\prime }$ expressing the vector of predictors (single‐nucleotide polymorphisms [SNPs], environmental covariates, etc.) for a candidate line. Note that $f\left(y_{c}|\theta ,{x^{\prime }}_{c}\right)p\left(\theta |y,X\right)$ is the kernel of the posterior predictive distribution (Gelman et al., 2014).

$$\;{\bar{L}}_{c}=\int_{y_{c}\in Y}\int_{\theta \in \Theta }L\;\left(F_{Y_{c}},\theta \right)f\left(y_{c}|\theta ,{x^{\prime }}_{c}\right)p\left(\theta |y,X\right)\partial \theta dy_{c}.$$

In our R package, the FastMPS and EvalMPS functions approximate this integral using MCMC samples of model parameters (here denoted as θ) from the BGLR R package, specifically from Multitrait function. A full description of Multitrait function and algorithms used can be consulted in Pérez‐Rodríguez and de los Campos (2022). Previous works (Villar‐Hernández et al., 2018, 2021) offer a comprehensive description of selection from the perspective of BDT.

In the case where multitrait regression is solved from a frequentist perspective or when only point estimates of predictions are used for each candidate line, the previous expression can be approximated by assuming multivariate normality of the predictive distribution. The approximation of the expected loss can be obtained through the ApproxMPS function.

Indeed, in the previous expression, we can replace the LF with any other function of choice. The MPS R package utilizes the EnergyS function and the MALF function as alternatives to the KL function. This flexibility permits the exploration and comparison of different LFs in the selection process.

### 2.3 Similarity and distance between lines

In the GS context, we can combine the theory of LFs with similarity or distance concepts. Suppose we have n candidate individuals for which there are k SNPs, given as an n×k incidence matrix X. The rows represent the candidates and columns represent the SNPs. We define the complete SNP profile as $x_{i}$ for individual i.

To define similarity between rows of X, we introduce a function S:X×X→R with the following assumptions (Ickstadt et al., 2005):

1. $S\left(x_{k},x_{l}\right)>S\left(x_{0},x_{l}\right)$ if $x_{l}$ is more similar to $x_{k}$ than to $x_{0}$, $x_{k}\neq x_{0};x_{k},x_{l},x_{0}\in X$; this assumption is known as comparability,
2. $S\;\left(x_{i},x_{j}\right)=\;S\left(x_{j},x_{i}\right)$, i,j=1,…,n, assumption of symmetry,
3. $S\left(x_{i},x_{j}\right)\geq S\left(x_{i},x_{i}\right)$, i,j=1,…,n, assumption of natural order,
4. $S(x_{i},x_{j})\geq 0$, i,j=1,…,n, assumption of positivity,
5. $S\;(x_{i},x_{i})=\;1$, i=1,…,n, assumption of normality.

A simple metric that fulfills the mentioned assumptions is the “simple matching coefficient” included in the MPS package within the SimMatrix function. This function takes a candidate's SNP matrix as an argument and returns a similarity matrix of dimension n×n. In the following example, we have four candidate lines for selection (rows), each with information on eight positions (columns). The similarity matrix is shown below, where the diagonal elements are all ones because an individual's profile is perfectly identical to itself in every position. Outside of the main diagonal, the values represent the proportion of positions that match with other lines. For example, lines 1 and 3 are similar in 37.5% of the positions, while lines 2 and 4 are similar in 25% of the positions.

**Table:** 

set.seed(624) # for reproducibility								
X <‐ matrix(sample(c(‐1,0,1), 8*4, replace = TRUE), nrow = 4)								
print(X)								
	[,1]	[,2]	[,3]	[,4]	[,5]	[,6]	[,7]	[,8]
[1,]	‐1	0	1	‐1	0	0	0	‐1
[2,]	0	‐1	0	0	1	1	0	0
[3,]	1	‐1	‐1	‐1	‐1	0	0	1
[4,]	0	‐1	‐1	1	‐1	0	1	‐1
sm <‐ SimMatrix(X) # similarity matrix								
print(sm)

**Table:** 

	[,1]	[,2]	[,3]	[,4]
[1,]	1.000	0.125	0.375	0.25
[2,]	0.125	1.000	0.250	0.25
[3,]	0.375	0.250	1.000	0.50
[4,]	0.250	0.250	0.500	1.00
print(AveSim(sm)) # average similarity				
[1] 0.2500000 0.2083333 0.3750000 0.3333333				

By taking the average with AveSim, the average similarity per candidate line is obtained. This average similarity can serve as an indicator for selecting individuals who, on average, are less related to the rest and also have the lowest expected loss based on the chosen LF.

Another approach to measure similarity between candidates is using distance measures. The AveDist function of MPS takes a matrix Xcand of dimension n×k and produces a distance matrix, using a method defined in the measure argument of this function. Allowed methods are “euclidean,” “maximum,” “manhattan,” “canberra,” “binary,” or “minkowski,” as in the base R distance function dist.

## 3 RESULTS

In this section, we introduce some examples of how to use the MPS R package with different multitrait regression models and datasets. Each simple example contains reproducible code snippets demonstrating the functionality of the MPS package.

### 3.1 Example 1: Multitrait GS

In this example, we use the wheat database that is included with the R package BGLR. The database consists of genotypic and phenotypic information of 599 wheat lines in four traits. The genotypic information comprises 1279 SNP‐type molecular markers. In the following code, the R memory is cleaned, and the working directory is fixed. After that the two R packages used are loaded into R (BGLR for regression model, MPS for evaluation of PEL). Y contains the phenotypic records and X contains the standardized genotypic data (SNP molecular markers).

**Table:** 

rm(list = ls())	# Cleaning R memory
setwd(“path in your computer”)	# Working directory
library(MPS); library(BGLR)	# Needed R Packages
data(wheat)	# Loading dataset
Y <‐ as.matrix(wheat.Y)	# Phenotypic records
X <‐ scale(wheat.X, center = TRUE, scale = TRUE) # Genotypic data	

After that, the data are partitioned, with 60% being used to train the model for prediction purposes (simulating the scenario of having related historical information), while the remaining 40% of the data simulate the set of candidate individuals for selection. set.seed(647) is used for reproducibility of results presented in this article. The ID of candidate/parental lines is sampled without replacement and then sorted and saved in the vector idPar. The last three lines of code perform a subsetting of phenotypic and genotypic data in the training and parental set.

**Table:** 

set.seed(647)	# Seed for reproducibility
n <‐ nrow(Y)	# Number of records
porc_parental <‐ 0.4	# Percentaje in candidates
# Random observation for candidate set	
idPar <‐ sort(sample(1:n, ceiling(porc_parental*n), replace = FALSE))	
XTrn <‐ X[‐idPar,]	# Genotypic data in Training set
XPar <‐ X[idPar, ]	# Genotypic data in Parental set
YTrn <‐ Y[‐idPar,]	# Phenotypic data in Training set

Next, the linear predictor is specified in the ETA object, imposing Gaussian priors (Bayesian ridge regression [BRR]) on regression coefficients to emulate Ridge Regression. The multitrait model is fitted using the Multitrait function of BGLR R package passing as arguments the phenotypic records and the linear predictor. Note that saveEffects = TRUE in both the linear predictor specification (ETA) and in the resCov argument in order to save posterior MCMC samples of regression coefficients and residual variance–covariance, respectively.

**Table:** 

# Specification of the linear predictor for BGLR
ETA <‐ list(list(X = X, model = "BRR", saveEffects = TRUE))
# Run the model using Multitrait function of BGLR R Package
model <‐ Multitrait(y = YTrn, ETA = ETA, intercept = TRUE,
resCov = list(type = "UN", saveEffects = TRUE),
nIter = 100000, burnIn = 30000)

The following lines of code illustrate how to read the posterior samples from the MCMC of the model's parameters. It is necessary to verify that the MCMC samples have converged to their stationary distributions (the CODA package of Plummer et al. [2006] can assist). After the aforementioned step, we pass these MCMC samples as input to the FastMPS function. Detailed explanations of each input passed to FastMPS function are shown below.

Xcand = XPar corresponds to the matrix of predictors (scaled incidence matrix based on SNPs) for the candidate lines of selection. The dimension of XPar is of *n* = 240 rows (candidates) and *k* = 1279 columns (predictors). B0 is a matrix of dimension *M* = 10,000 × *t* = 4 corresponding to the intercept/overall mean term in the linear model, where M is the number of MCMC samples after burn‐in and t is the number of traits. The next argument is B, an array containing regression coefficients of dimension *M* = 10,000 × *k* = 1279 × *t* = 4 of MCMC samples. R input represents a matrix of dimension M×(t×(t+1)/2) of MCMC samples of the variance–covariance components in the residual covariance matrix. In this example, R matrix has dimensions 10,000 × 10. In the last argument of FastMPS function, the method is set as “kl” for KL LF. The last line of code obtains the average similarity between each line with the remaining candidates.

**Table:** 

# Reading Posterior MCMC of model parameters to compute expected loss
B0 <‐ as.matrix(read.table(file = "mu.dat", # Overall mean
header = FALSE))
B <‐ readBinMatMultitrait(‘ETA_1_beta.bin’) # Regression coefficients
R <‐ as.matrix(read.table(file = “R.dat”, # Residual covariance matrix
header = FALSE))

**Table:** 

# Evaluate loss function
out <‐ FastMPS(Xcand = XPar, B0 = B0, B = B, R = R, method = “kl”)
# Get average distances for each candidate
s <‐ AveSim(SimMatrix(wheat.X[idPar,]))

After running the FastMPS function, the output list contains the PEL (loss), the ranking of each candidate line for selection, and a data.frame (yHat) containing the predictions of BVs for all traits.

**Table:** 

> str(out)
List of 4
$ method : chr "kl"
$ loss : num [1:240] 0.482 0.466 0.222 0.182 0.49 …
$ ranking: num [1:240] 214 208 71 41 217 236 201 59 126 158 …
$ yHat :‘data.frame’: 240 obs. of 4 variables:
..$ X1: num [1:240] ‐0.833 ‐0.847 0.497 0.511 ‐0.125 …
..$ X2: num [1:240] 0.186 0.139 ‐0.394 0.211 ‐0.348 …
..$ X3: num [1:240] ‐0.366 ‐0.4283 ‐0.2819 0.1197 0.0116 …
..$ X4: num [1:240] ‐0.276 ‐0.2762 ‐0.3616 ‐0.0803 0.0753 …
‐ attr(*, "class")= chr "MPS"

**FIGURE 1:**
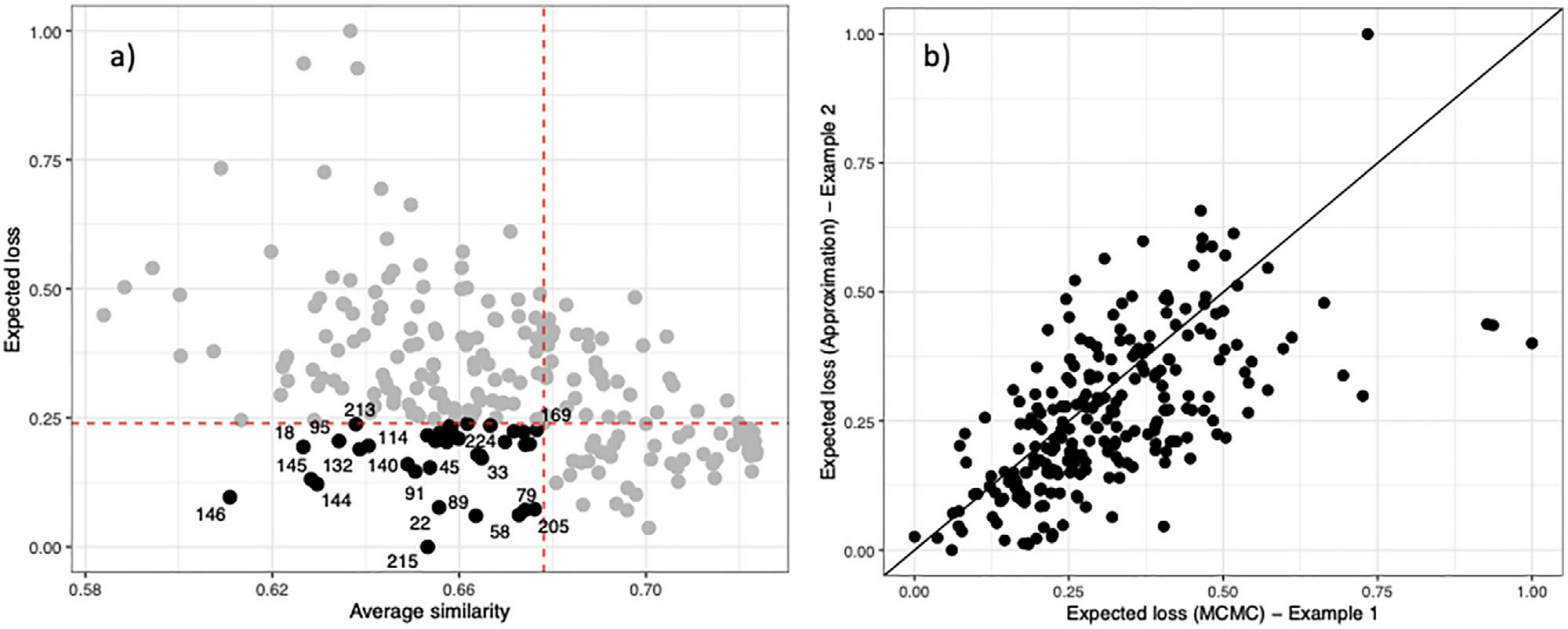
(a) The average similarity of each candidate with the rest is given on the *x*‐axis, while the *y*‐axis gives the posterior expected loss for each candidate. The filled black circles indicate candidates with the minimum posterior expected loss and minimum similarity, as shown in Example 1. (b) A comparison of the expected loss through MCMC in Example 1 (genomic data only) and the approximate expected loss in Example 2 (combining genomic and pedigree data). The linear Pearson correlation coefficient between them is 0.63.

In Figure 1a, results from this example are shown. Similarity values for candidate individuals are represented on the *x*‐axis, while expected loss is represented on the *y*‐axis. Vertical dashed lines indicate the 35th percentile of expected losses, and horizontal dashed lines indicate the 65th percentile of similarity. Different configurations can be tested for the maximum expected loss value and the maximum similarity value until the desired number of individuals for selection is reached.

A breeder may prefer those lines that, on average, are dissimilar from the rest. By combining information on the PEL and similarities, it is possible to select a subset of individuals that exhibit strong performance (i.e., minimal PEL) across multiple traits while also maximizing their divergence from other lines (i.e., promoting diversity). This approach permits the identification of lines that exhibit both desirable trait performance and distinct genetic characteristics.

### 3.2 Example 2: Multitrait genomic + pedigree selection

For this example, the same database used in the previous exercise (Example 1) is employed, but with the incorporation of pedigree and molecular markers as predictors. The first part of the code is very similar to that of Example 1, so we omit its explanation. The only difference here is that the pedigree information is stored in the matrix K.

**Table:** 

rm(list = ls())	# Cleaning memory
setwd("path in your computer")	# Working directory
library(MPS); library(BGLR)	# Needed R Packages
data(wheat)	# Loading dataset

**Table:** 

Y <‐ as.matrix(wheat.Y)	# Phenotypic records
X <‐ scale(wheat.X, center = TRUE, scale = TRUE) # Genotypic data	
K <‐ wheat.A	

As in the previous example, 60% of the data are allocated for training the model, while the remaining 40% simulate the role of candidate lines for selection.

**Table:** 

set.seed(647)	# Seed for reproducibility
n <‐ nrow(Y)	# Number of records
porc_parental <‐ 0.4	# Percentage in candidates
# Random observation for candidate set	
idPar <‐ sort(sample(1:n, ceiling(porc_parental*n), replace = FALSE))	
YTrn <‐ Y; YTrn[idPar,] <‐ NA	

The linear predictor now incorporates the matrix of molecular markers, and associated regression coefficients are subject to L2‐norm penalization (Ridge). Meanwhile, the pedigree matrix (K) is used to incorporate random effects, which are modeled through nonparametric genomic regressions based on Reproducing Kernel Hilbert spaces (RKHS) methods; details of RKHS approach can be found in Pérez‐Rodríguez and de los Campos (2022).

**Table:** 

# ModelFit using BGLR
ETA <‐ list(list(X = X, model = “BRR”), list(K = K, model = “RKHS”))
model <‐ Multitrait(y = YTrn, ETA = ETA, intercept = TRUE,
resCov = list(type = “UN”), nIter = 100000, burnIn = 30000)

Finally, outputs from Multitrait R function are inputs of the MPS R package. In this example, the ApproxMPS function is used, which is a version that approximates the loss of each candidate line for selection when the MCMC samples are not available (NA) from BGLR. This approximation should be taken with caution, as it assumes that the predictive distribution of each candidate line is multivariate normal.

The ApproxMPS function receives several arguments. First, B0 is a vector representing the overall mean of each trait, with a length equal to the number of traits (*t* = 4). yHat is the matrix of punctual posterior means for each candidate, with dimensions *n* = 240 × *t* = 4, where *n* is the number of candidates and *t* is the number of traits. The next argument is R, which is a square matrix representing the residual covariance matrix with dimensions *t* = 4 × *t* = 4.

The target argument is an optional vector of length equal to the number of traits (*t* = 4) that reflects the breeder's expectation. If not provided, the algorithm determines it internally. Lastly, the method argument specifies the LF to be used and must be one of “kl” for KL, “energy” for EnergyS, or “malf” for Multivariate Asymmetric Loss. The KL functions is used here.

**Table:** 

B0 <‐ as.numeric(model$mu)	# Overall mean
yHat <‐ model$ETAHat[model$missing_records,]	# Puntual BVs
R <‐ as.matrix(model$resCov$R)	# Residual Covariance matrix
out <‐ ApproxMPS(B0 = B0, yHat = yHat,	# Evaluate loss functions
R = R, method = "kl")	

The ApproxMPS function returns a list that is the same as the output of the FastMPS or EvalMPS functions. The expected loss solely or combined with average similarity of the lines can be combined to identify individuals that ensure both maximum genetic progress and diversity. A natural question is how expected loss is similar in both approaches (full MCMC vs. approximation)? In Figure 1b, it can be observed that the expected losses in both approaches exhibit a positive linear correlation (correlation coefficient of 0.63). It is important to highlight that as the predictive distributions of each candidate deviate from multivariate normality, the expected loss obtained with ApproxMPS will differ from the expected loss obtained with FastMPS.

### 3.3 Example 3: Multitrait selection with positive and negative direction of genetic progress

In this example, we employed a database named AdvEYT, which is included in the MPS R package. The data comprised phenotypic records for 190 lines for 11 traits: GY_B5IR_F5IR_BEHT, GY_BLHT, GY_B2IR, and GY_TPE belonging to the grain yield category; Zinc, TKW, GRNPRO, and ALVW fall under the quality category; and YR_LUD, YR_NJ, and SR_NJ correspond to the diseases category. Furthermore, the dataset includes information on 8545 SNP‐type molecular markers.

The objective of this analysis is to identify the top 15% of lines (approximately 28 lines) based on the criterion of minimum expected loss. For the Grain Yield and Quality traits, the desired direction of genetic progress is positive, whereas for the Diseases category traits, progress in the negative direction is sought.

The first code segment clears the R memory, sets the desired working directory, imports the necessary libraries, and loads the database to be used. For computational convenience, we use the genomic relationship matrix ($G\;=\frac{{\mathrm{ZZ}}^{\prime }}{p}\;$) to fit a multitrait Genomic Best Linear Unbiased Prediction (GBLUP) model, where Z is the standardized SNP matrix. The last line creates a copy of the phenotypic records matrix to reverse direction of observed phenotypic values for Disease category traits.

**Table:** 

rm(list = ls());	# Clean R memory
setwd(“your‐path”)	# Set the working directory
library(MPS); library(BGLR)	# Load R Packages
data(AdvEYT)	# Load data
Z <‐ scale(X, center = TRUE, scale = TRUE)	# Standardized SNPs
G <‐ tcrossprod(Z) /ncol(Z)	# G relationship matrix
Y2 <‐ Y	# copy of the response

Next, we define a vector that indicates the desired direction of improvement: 1 indicates that we aim to increase the genetic value in that trait, while −1 indicates a desire to decrease the genetic value. In this case, first eight traits in the phenotypic records are traits that we aim to increase, while the final three traits are ones we aim to decrease; therefore, the direction object has eight ones and finally three minus ones. Subsequently, in Y2, we reverse the direction of the response variable accordingly. We define the linear predictor (ETA) as a function of the G matrix by incorporating random effects, which are modeled through nonparametric genomic regressions based on RKHS methods. This approach allows BGLR to achieve computationally efficient model fitting; however, it does not yield MCMC chains. Therefore, we will use the ApproxMPS function from the MPR package to approximate expected losses. Note that Y2 is given to the regression model using the Multitrait function from BGLR, not Y.

**Table:** 

direction <‐ c(1,1,1,1,1,1,1,1,‐1,‐1,‐1)
Y2 <‐ sweep(Y2, 2, direction, ‘*’)
ETA <‐ list(list(K = G, model = "RKHS"))
model <‐ Multitrait(y = Y2, ETA = ETA, intercept = TRUE,
resCov = list(type = "UN"),
nIter = 100000, burnIn = 20000)

Finally, the ApproxMPS function is called, and arguments similar to those used in Example 2 are provided, with these being the outputs of BGLR.

**Table:** 

out <‐ ApproxMPS(B0 = as.numeric(model$mu), yHat = model$ETAHat,
R = as.matrix(model$resCov$R), method = "kl",
direction = direction)

**FIGURE 2:**
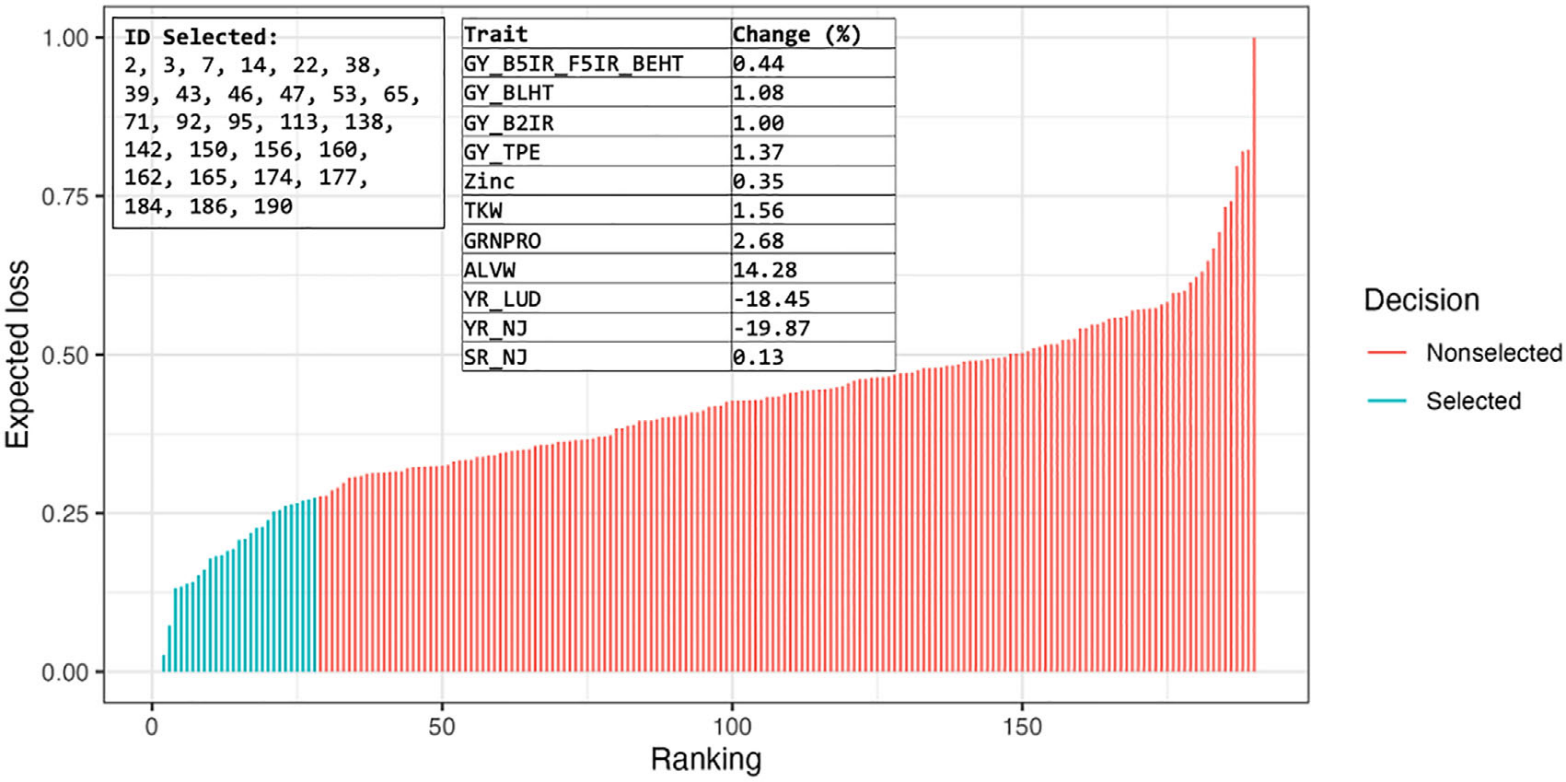
Ranking of the 190 lines according to the expected loss criterion. The IDs of the 28 selected lines are presented in the first table. The expected change (percentage) in the average breeding values (estimated by the model) of the selected lines compared to the nonselected ones by trait is presented in the second table.

In Figure 2, the ranking of each candidate based on expected loss is displayed. The selected lines are those that rank between 1 and 18, highlighted in turquoise blue. The first table in Figure 2 displays the IDs of the selected lines, while the second table reports the percentage change in average BVs (estimated by the model) between selected and nonselected lines, calculated as [(${\bar{\mathrm{BV}}}_{\mathrm{sel}}-{\bar{\mathrm{BV}}}_{\mathrm{nonsel}}$)/${\bar{\mathrm{BV}}}_{\mathrm{nonsel}}\times$100], where ${\bar{\mathrm{BV}}}_{\mathrm{sel}}$ represents average BVs of selected lines, while ${\bar{\mathrm{BV}}}_{\mathrm{nonsel}}$ is the average BVs of nonselected lines.

It is worth noting that there are significant changes in average BVs for certain traits, particularly for traits within the Disease category, with changes as large as −18.45% for YR_LUD and −19.87% for YR_NJ, and a 14.28% change for the ALVW trait within the quality category. The remaining traits exhibit relatively minor differences.

### 3.4 Example 4

One of the arguments of the functions FastMPS, EvalMPS, and ApproxMPS is target, which can be used by the researcher to indicate their objectives in the selection to the software. In this example, we used the AdvEYT database that accompanies our package. We only employed three traits: GY_B5IR_F5IR_BEHT, GRNPRO, and YR_LUD. GY_B5IR_F5IR_BEHT and GRNPRO have a linear correlation of −0.3; GY_B5IR_F5IR_BEHT and YR_LUD have a correlation of 0.23; and GRNPRO and YR_LUD have a correlation close to 0.

**FIGURE 3:**
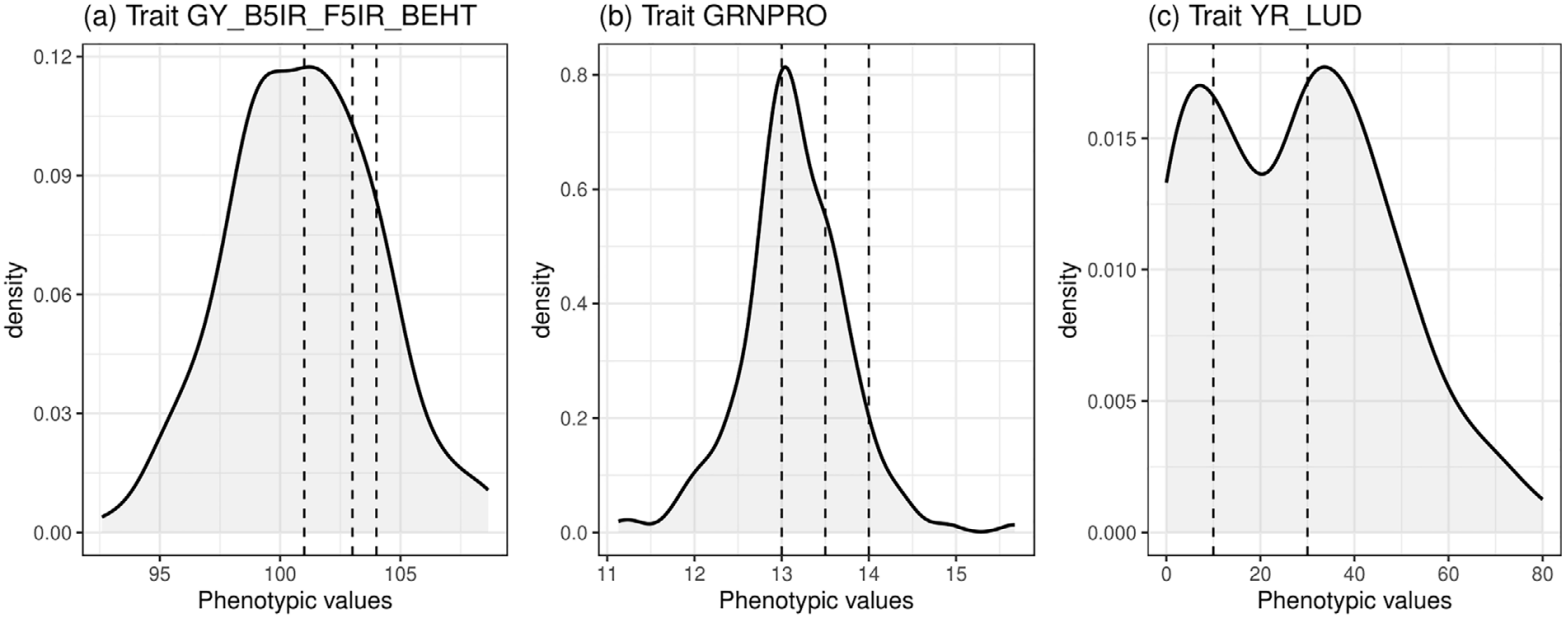
Distributions of traits (a) GY_B5IR_F5IR_BEHT, (b) GRNPRO, and (c) YR_LUD. Dashed vertical lines indicate different target values for each trait.

Figure 3a–c displays the distributions of the traits. It can be noted that GY_B5IR_F5IR_BEHT and GRNPRO have approximately symmetric distributions, whereas YR_LUD exhibits an asymmetric and bimodal distribution, making the selection process more complex.

The dashed lines represent different target values for each trait. In the case of the trait YR_LUD, which represents a disease, the direction of improvement is decreasing. Thus, we set two target values (scenarios): 30 and 10 (percentiles 25 and 50, respectively). For the trait GY_B5IR_F5IR_BEHT, we set three desired targets (scenarios): 101, 103, and 104 (percentiles 50, 75, and 90, respectively). As for the trait GRNPRO, the selection targets (scenarios) are 13, 13.5, and 14 (percentiles 50, 75, and 90, respectively). Subsequently, we have created 18 possible scenarios resulting from the combinations of the targets, summarized in Table S1.

**FIGURE 4:**
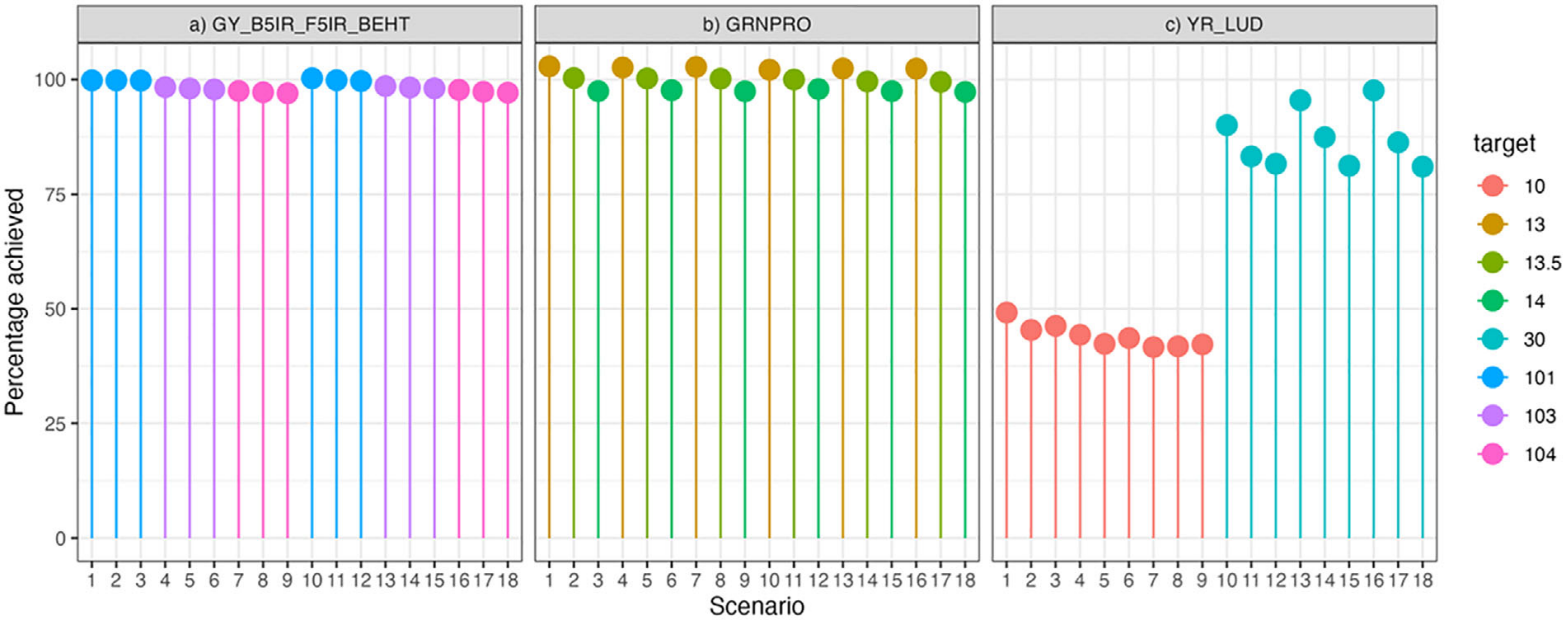
Percentage achieved by average breeding values with respect to the target value in each scenario (Table S1) for the three traits under selection.

After fitting the multitrait regression model as in Example 1, the expected loss for each candidate line was obtained, and the 20 lines with the lowest expected loss were chosen. Once the selection was made, the BVs of the selected lines were averaged. After that, the percentage achieved of average BVs with respect to the target value was calculated, which is shown in Figure 4a–c.

For the traits GY_B5IR_F5IR_BEHT and GRNPRO, the realized average BVs were generally close to, and sometimes more than, 100% of the target BVs. In the case of the trait YR_LUD, when the target was set at 30, the average BVs reached on average 87% with respect the target, with minimum 80% and maximum 97% (remember that for this trait, improvement is desired in the negative direction, and smaller BVs are better). In the worst‐case scenario (target = 10), average BVs reached on average 22.7%, with minimum value equal to 20.3% and maximum value equal to 24%. This could be due to the bimodal distribution of this trait or because around 22 units lies what is known as the Pareto frontier for this trait.

### 3.5 Example 5: Candidates’ selection with missing phenotypic records for some traits

At times, only partial phenotypic information is available for certain traits in some candidate lines for selection. By combining prediction using BGLR and expected loss calculation using MPS, it is possible to identify which lines the breeder should select. To illustrate this concept, the database accompanying the MPS package is used. Let us assume we are interested in identifying the top 20% (40 lines) and considering four traits: GY_BLHT, TKW, GRNPRO, and SR_NJ. However, out of the 190 lines in the dataset, 70 of them lack phenotypic records for the trait GY_BLHT, 50 lines have no information on TKW, 60 lines have missing data for GRNPRO, and 20 lines have missing information for SR_NJ.

The following set of code clears the R memory, sets the working directory, loads the necessary packages, generates a seed for reproducibility of this example, and subsets the relevant traits.

**Table:** 

rm(list = ls())	# Clean Work space
setwd(“your‐path”)	# Working directory
library(MPS); library(BGLR)	# Needed libraries
set.seed(752); data(AdvEYT)	# Reproducibility and Load dataset
Y <‐ Y[,c(‘GY_BLHT’, ‘TKW’, ‘GRNPRO’, ‘SR_NJ’)]	# Traits of interest
n <‐ nrow(Y)	# Number of candidates

To simulate the scenario of missing data, randomly missing data (NA) are introduced. As improvement direction is decreasing for the third trait, it is multiplied by −1.

**Table:** 

# Allocating randomly missing phenotypic data in each trait
Y[sort(sample(1:n, size = 70, replace = FALSE)), 1] <‐ NA
Y[sort(sample(1:n, size = 50, replace = FALSE)), 2] <‐ NA
Y[sort(sample(1:n, size = 60, replace = FALSE)), 3] <‐ NA
Y[sort(sample(1:n, size = 20, replace = FALSE)), 4] <‐ NA
Y2 <‐ Y # Copy of phenotypic records
Y2[,4] <‐ Y2[,4] * ‐1 # Change sign in SR_NJ trait

The genotypic information is standardized, and the linear predictor (ETA) is defined by selecting model = SpikeSlab to instruct BGLR to impose prior distributions on regression coefficients, allowing for automatic variable selection (for details, see Pérez‐Rodríguez & de los Campos, 2022). MCMC chains are saved via the argument saveEffects = TRUE. Finally, the model is fitted by specifying the number of iterations (nIter) and the burn‐in (burnIn). The argument resCov = list(type = “UN,” saveEffects = TRUE) indicates to BGLR that an unstructured covariance matrix is assumed for the residual covariance matrix of the linear model, and it is also requested to save the MCMC chains for this matrix.

**Table:** 

# Center and scaling genomic info
X <‐ scale(X, center = TRUE, scale = TRUE)
# Specification of the linear predictor for BGLR
ETA <‐ list(list(X = X, model = "SpikeSlab", saveEffects = TRUE))
# Run the model
model <‐ Multitrait(y = Y2, ETA = ETA, intercept = TRUE,
resCov = list(type = "UN", saveEffects = TRUE), nIter = 100000, burnIn = 30000)

To obtain the expected loss, it is necessary to read the MCMC chains from the fitted model, which serve as input to the FastMPS function. The direction argument specifies that for the first three traits, the genetic value needs to be increased, therefore appearing three ones, while for the fourth trait, the progress is in the opposite direction, appearing a minus one. Finally, the average values of the predicted BVs for the selected and nonselected lines are presented.

**Table:** 

# Reading Posterior MCMC of model parameters & direction
B0 <‐ as.matrix(read.table(file = "mu.dat", header = FALSE)) # Overall mean
B <‐ readBinMatMultitrait(‘ETA_1_beta.bin’) # Regression coefficients
R <‐ as.matrix(read.table(file = "R.dat", header = FALSE)) # Residual cov. matrix
direction <‐ c(1,1,1,‐1) # Direction of improvement
out <‐ FastMPS(Xcand = X, B0 = B0, B = B, R = R, # Evaluation of Loss Function
method = "kl", direction = direction)
idToSelect <‐ out$ranking %in% 1:40 # ID of selected
yHat <‐ out$yHat # Predicted BVs
yHat[,4] <‐ ‐1*yHat[,4] # Reverse order of BVs for trait 4
colMeans(yHat[idToSelect,]) # Average BVs of selected
X1 X2 X3 X4
101.85855 53.29620 13.35975 22.59924
colMeans(yHat[!idToSelect,]) # Average BVs of non‐selected
X1 X2 X3 X4
100.95011 52.45389 13.10432 26.27064

The IDs of the selected individuals are presented below. The number in parentheses indicates the count of missing trait records. For instance, “1(3)” denotes that the top line (with the minimum expected loss) is the first candidate in the dataset, which originally had three missing traits records. The same explanation applies to the rest of the selected candidates. This example illustrates how breeders can effectively choose individuals even in the presence of missing phenotypic records for some traits.

**Table:** 

NAsBySelLines <‐ apply(Y2[which(idToSelect),], 1, function(x) sum(is.na(x)))
IDSel <‐ which(idToSelect)
print(paste0(IDSel, ‘(‘, NAsBySelLines, ’)’))
[1] "1(3)" "4(2)" "14(1)" "21(4)" "29(1)" "34(2)" "40(2)" "42(3)"
[9] "49(1)" "50(3)" "53(1)" "54(2)" "58(3)" "63(3)" "65(2)" "66(1)"
[17] "70(3)" "73(1)" "79(2)" "92(1)" "95(0)" "99(1)" "101(3)" "118(2)"
[25] "119(1)" "129(0)" "131(3)" "137(1)" "142(3)" "143(2)" "144(2)" "147(1)"
[33] "148(3)" "149(1)" "160(1)" "162(2)" "171(2)" "173(2)" "174(3)" "190(2)"

### 3.6 Example 6: Identifying superior candidates in multi‐environment

Sometimes, we have records for a specific trait assessed in different individuals and environments. MPS can also assist in identifying, from a set of candidates, which individuals perform best across all environments. In this example, we have taken data from 250 lines evaluated in three environments (“Bed5IR,” “Drip,” and “Bed2IR”) included in MPS. The original data, labeled “DATASET4to5.Wheat.rdata,” can be accessed at https://hdl.handle.net/11529/10907, and its full description can be found in Montesinos‐López et al. (2017).

The R code for this example is in Code S1‐Example 6 in the Supporting Information. To borrow information across environments while allowing marker effects to change across environments, we fitted a Markers–Environment interaction model (M×E GBLUP) (López‐Cruz et al., 2015). Detailed explanation how to fit M×E GBLUP model using BGLR can be found in https://github.com/MarcooLopez. To simulate scenarios of Training and Candidate/Parental sets, the data were randomly split into 20% (50 lines) for selection candidates and 80% as training data. After fitting the model and approximating the expected loss for every candidate, we retained the top 40% of candidates (20 lines) with the minimum expected loss.

**FIGURE 5:**
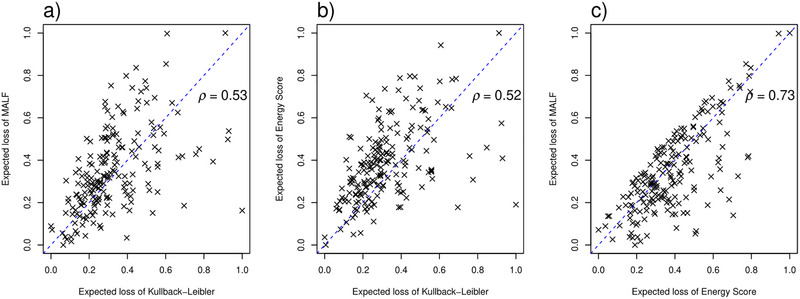
Difference between the average breeding values of the selected lines compared to the overall mean (across the three environments, estimated by the model) and the means within each environment (estimated by the model).

Figure 5 shows the difference between the average BVs of the selected lines compared to the overall mean (across the three environments, estimated by the model) and the means within each environment (estimated by the model). It is worth noting that in all cases, there is a difference of more than 9 units compared to the overall mean and more than 2 units compared to the means within each environment. In other words, the selected lines exhibit superior performance in each individual environment as well as across all environments. If these lines become the parents of the next generation, it is expected that the average genetic superiority will be passed on to the next generation.

### 3.7 Linear correlation between loss functions

**FIGURE 6:**
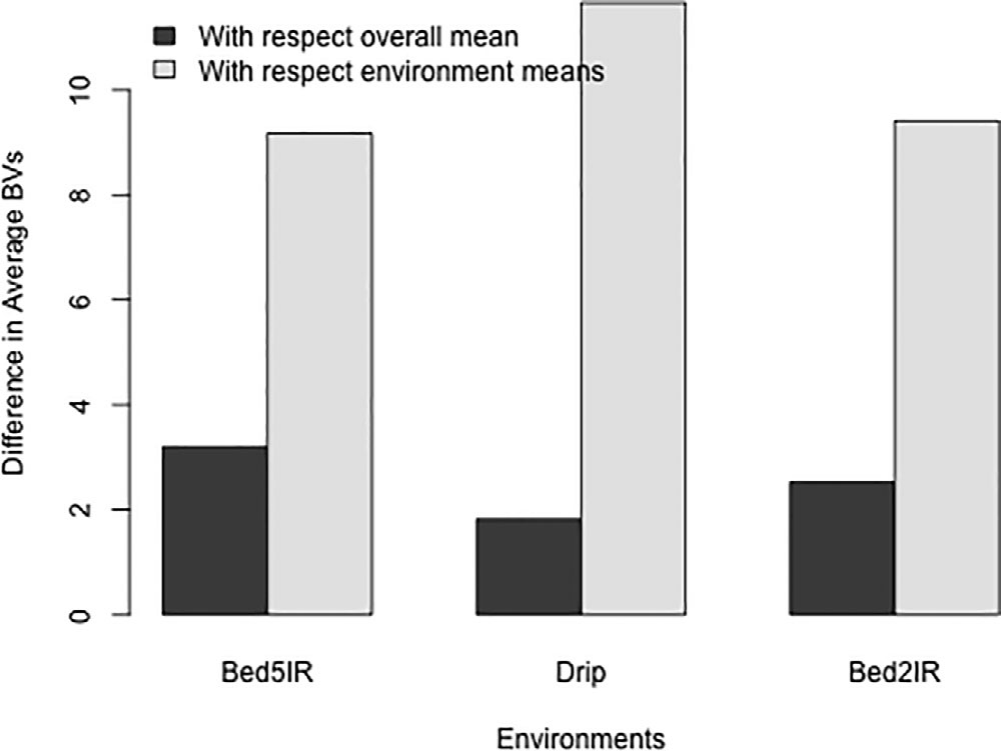
Linear relationship between expected loss in three loss function comparisons: (a) Kullback–Leibler versus MALF, (b) Kullback–Leibler versus Energy Score, and (c) Energy Score versus MALF for expected losses in Example 5.

In the Example 6, when transitioning from the kl LF to the other two alternatives, namely, energy and malf, it becomes possible to quantify the extent of linear association between expected loss values using the Pearson correlation coefficient. Notably, the KL LF demonstrates a comparable level of association with the MALF (ρ=0.53) and EnergyS functions (ρ=0.52). However, the MALF and EnergyS functions had the most robust linear association (ρ=0.73) (Figure 6a–c). It is important to note that these findings may vary depending on the specific database employed.

## 4 DISCUSSION

In conventional phenotypic selection and GS, the decisions taken to select and mate parents to form an improved population are crucial. However, when improving the economic value of a crop, plant breeding programs consider multiple traits simultaneously, as economic value and genetic merit depend on more than one trait. When traits are correlated, either positively or negatively, the criterion for selecting the best individuals in a population becomes more complex.

Creating software for the selection of parents in genomics provides a powerful tool for genetic improvement, precision breeding, conservation genetics, and resource optimization. It enhances the efficiency, accuracy, and speed of breeding programs, leading to improved outcomes in agriculture, animal breeding, conservation efforts, and so forth.

In this article, we presented the MPS R package software to facilitate the multitrait selection of parental selection, primarily in GS, in animal and plant breeding. The software utilizes the Bayesian optimization algorithm with three LFs for selecting parental candidates. We provided our proposal by presenting some simple application examples.

The MPS R package provides the functionality to perform multitrait selection based on the Bayesian optimization algorithm. The package includes three main functions: EvalMPS, FastMPS, and ApproxMPS. EvalMPS and FastMPS are suitable when all MCMC samples are available, while ApproxMPS relies only on point estimates. These functions take the necessary arguments, such as predictors of candidate individuals, regression coefficients, residual covariance matrices, and the choice of LF. The package also provides options for different LFs, including KL, EnergyS, and Multivariate Asymmetric Loss.

The theory of LFs applied in genetic gain selection programs is built upon the concept of selection by truncation in single traits and extends to multitrait selection. The KL LF is derived from the expected distance between the theoretical multivariate distribution under truncation and the multivariate distribution of a candidate line. The LF is further approximated using MCMC samples from the Bayesian multitrait linear models. The MPS package allows for the exploration and comparison of different LFs, enabling breeders to select the most suitable one for their specific needs.

To incorporate similarity or distance concepts, the MPS package includes the “simple matching coefficient” as a metric that fulfills the required assumptions of comparability, symmetry, natural order, positivity, and normality. The SimMatrix function generates a similarity matrix based on the candidate SNP matrix, representing the proportion of positions that match between individuals. The average similarity per candidate line can be obtained by taking the average of this matrix, excluding the main diagonal.

The presented examples demonstrate the functionality of the MPS package in multitrait GS. The software allows breeders to combine information on PEL and similarities to select a subset of individuals that exhibit strong performance across multiple traits while maximizing diversity. The results obtained from the examples show the identification of lines with minimal expected loss and maximum dissimilarity, enabling breeders to make informed decisions regarding parental selection.

## 5 CONCLUSION

In this study, we introduce the MPS R package, a valuable tool for enhancing multitrait genetic improvement in both phenotypic selection and GS. Our software leverages Bayesian optimization algorithms in conjunction with the KL, the EnergyS, and the Multivariate Asymmetric Loss as LFs for the selection of parental candidates. Additionally, the MPS R package incorporates the “simple matching coefficient” to evaluate similarities between individuals. By amalgamating information on expected loss and similarities, breeders can choose individuals with robust performance across multiple traits while maximizing diversity.

The application of the software is illustrated through straightforward examples accompanied by code snippets in the R programming language. The MPS R package is intricately designed to seamlessly integrate with the popular BGLR R package, which offers a wide array of Bayesian multitrait regression models in GS.

The primary goal of the MPS R package is to provide the breeding community with software that enhances breeding efficiency and accuracy, ultimately leading to improved outcomes in agriculture and animal breeding, among other fields, by selecting individuals that fulfill multiple trait objectives.

## AUTHOR CONTRIBUTIONS

**Bartolo de J. Villar‐Hernández**: Conceptualization; formal analysis; investigation; software; writing—original draft; writing—review and editing. **Susanne Dreisigacker**: Project administration; resources; validation; writing—review and editing. **Leo Crespo**: Conceptualization; data curation; methodology; software; writing—review and editing. **Paulino Pérez‐Rodríguez**: Conceptualization; methodology; software; writing—review and editing. **Sergio Pérez‐Elizalde**: Investigation; software. **Fernando Toledo**: Conceptualization; software; validation; writing—review and editing. **José Crossa**: Conceptualization; investigation; methodology; writing—original draft; writing—review and editing.

## CONFLICT OF INTEREST STATEMENT

The authors declare no conflicts of interest.

## Supporting information

Supplementary Information

Supplementary Information

## Data Availability

The data can be found in each of the examples described and used in this study.
