# Supplementary material for: A Bayesian optimization R package for multitrait parental selection
Source: Plant Genome. 2024 Feb 22;17(2):e20433. doi: 10.1002/tpg2.20433 (PMC12806911; doi:10.1002/tpg2.20433)
Supplement: Supplementary file 1 [file TPG2-17-e20433-s002.docx]

**Supplementary Figure S1. Summarized workflow for using Bayesian Decision Theory within the MPS R Package.**

**Supplementary Table S1. Scenarios generated by the combination of different targets for each trait.**

| **SCENARIO** | **YR_LUD** | **GRNPRO** | **GY_B5IR_F5IR_BEHT** |
| --- | --- | --- | --- |
| 1 | 10 | 13 | 101 |
| 2 | 10 | 13.5 | 101 |
| 3 | 10 | 14 | 101 |
| 4 | 10 | 13 | 103 |
| 5 | 10 | 13.5 | 103 |
| 6 | 10 | 14 | 103 |
| 7 | 10 | 13 | 104 |
| 8 | 10 | 13.5 | 104 |
| 9 | 10 | 14 | 104 |
| 10 | 30 | 13 | 101 |
| 11 | 30 | 13.5 | 101 |
| 12 | 30 | 14 | 101 |
| 13 | 30 | 13 | 103 |
| 14 | 30 | 13.5 | 103 |
| 15 | 30 | 14 | 103 |
| 16 | 30 | 13 | 104 |
| 17 | 30 | 13.5 | 104 |
| 18 | 30 | 14 | 104 |

**Supplementary Code S1. R codes of the examples in “A Bayesian Optimization R package for Multi-trait Parental Selection”**

**System Information**

```
## - - - - - - - - - - - - - - - - - - - - - - - - - - - - - - - - ##
## Code to reproduce the examples from the article:
## A Bayesian Optimization R package for Multi-trait Parental Selection.
## Villar et. al. 2023
## - - - - - - - - - - - - - - - - - - - - - - - - - - - - - - - - ##
# > sessionInfo() # Useful Infos **Reproducible Research**
# R version 4.2.1 (2022-06-23)
# Platform: aarch64-apple-darwin20 (64-bit)
# Running under: macOS 14.0

# Matrix products: default
# LAPACK: /Library/Frameworks/R.framework/Versions/4.2-arm64/Resources/lib/libRlapack.dylib

# locale:
# [1] C/UTF-8/C/C/C/C

# attached base packages:
# [1] stats     graphics  grDevices utils     datasets  methods   base

# loaded via a namespace (and not attached):
# [1] compiler_4.2.1
```

**Example 1:**

```
## - - - - - - - - - - - - - - - - - - - - - - - - - - - - - - - - ##
##           Example 1
## - - - - - - - - - - - - - - - - - - - - - - - - - - - - - - - - ##

rm(list = ls())                  # Cleaning R memory
setwd("path in your computer")   # Working directory
library(MPS); library(BGLR)      # Needed R Packages
data(wheat)                      # Loading dataset

Y <- as.matrix(wheat.Y)          # Phenotypic records
X <- scale(wheat.X, center=TRUE, scale = TRUE) # Genotypic data

set.seed(647)             # Seed for reproducibility
n <- nrow(Y)              # Number of records
porc_parental <- 0.4      # Percentaje in candidates
idPar <- sort(sample(1:n, # Random observation for candidate set
                     ceiling(porc_parental*n),
                     replace = FALSE))

XTrn <- X[-idPar,]        # Genotypic data in Training set
XPar <- X[idPar, ]        # Genotypic data in Parental set
YTrn <- Y[-idPar,]        # Phenotypic data in Training set

# Specification of the linear predictor for BGLR
ETA <- list(list(X = X, model = "BRR", saveEffects = TRUE))

# Run the model using Multitrait function of BGLR R Package
model <- Multitrait(y = YTrn, ETA = ETA, intercept = TRUE,
                    resCov = list(type = "UN", saveEffects = TRUE),
                    nIter = 100000, burnIn = 30000)

# Reading Posterior MCMC of model parameters to compute expected loss
B0 <- as.matrix(read.table(file = "mu.dat",  # Overall mean
                           header = FALSE))
B <- readBinMatMultitrait('ETA_1_beta.bin')  # Regression coefficients
R <- as.matrix(read.table(file = "R.dat",    # Residual covariance matrix
                          header = FALSE))

# Evaluate loss function
out <- FastMPS(Xcand = XPar, B0 = B0, B = B, R = R, method = "kl")
out

# Get average distances for each candidate
s <- AveSim(SimMatrix(wheat.X[idPar,]))
```

**Example 2:**

```
## - - - - - - - - - - - - - - - - - - - - - - - - - - - - - - - - ##
##           Example 2
## - - - - - - - - - - - - - - - - - - - - - - - - - - - - - - - - ##
rm(list = ls())                  # Cleaning memory
setwd("path in your computer")   # Working directory
library(MPS); library(BGLR)      # Needed R Packages
data(wheat)                      # Loading dataset

Y <- as.matrix(wheat.Y)          # Phenotypic records
X <- scale(wheat.X, center = TRUE, scale = TRUE) # Genotypic data
K <- wheat.A

set.seed(647)             # Seed for reproducibility
n <- nrow(Y)              # Number of records
porc_parental <- 0.4      # Percentage in candidates
idPar <- sort(sample(1:n, # Random observation for candidate set
                     ceiling(porc_parental*n),
                     replace = FALSE))
YTrn <- Y; YTrn[idPar,] <- NA


# ModelFit using BGLR
ETA <- list(list(X = X, model = "BRR"), list(K = K, model = "RKHS"))
model <- Multitrait(y = YTrn, ETA = ETA, intercept = TRUE,
        resCov = list(type = "UN"), nIter = 100000, burnIn = 30000)

B0 <- as.numeric(model$mu)                    # Overall mean
yHat <- model$ETAHat[model$missing_records, ] # Puntual BVs
R <- as.matrix(model$resCov$R)                # Residual Covariance matrix
out <- ApproxMPS(B0 = B0, yHat = yHat,        # Evaluate loss functions
     R = R, method = "kl")

# Best 15% = 36 lines
selected_lines <- which(order(out$loss) %in% 1:36)
print(selected_lines)
```

**Example 3:**

```
## - - - - - - - - - - - - - - - - - - - - - - - - - - - - - - - - ##
##           Example 3
## - - - - - - - - - - - - - - - - - - - - - - - - - - - - - - - - ##
rm(list = ls());                                 # Clean R memory
setwd("your-path")                               # Set the working directory
library(MPS); library(BGLR)                      # Load R Packages
data(AdvEYT)                                     # Load data
Z <- scale(X, center = TRUE, scale = TRUE)       # Standardized SNPs
G <- tcrossprod(Z) /ncol(Z)                      # G relationship matrix
Y2 <- Y                                          # copy of the response


direction <- c(1,1,1,1,1,1,1,1,-1,-1,-1)
Y2 <- sweep(Y2, 2, direction, '*')
ETA <- list(list(K = G, model = "RKHS"))
model <- Multitrait(y = Y2, ETA = ETA, intercept = TRUE,
          resCov = list(type = "UN"),
          nIter = 100000, burnIn = 20000)

out <- ApproxMPS(B0 = as.numeric(model$mu), yHat = model$ETAHat ,
                 R = as.matrix(model$resCov$R), method = "kl",
                 direction = direction)

# Best 15% = 30 lines
selected_lines <- which(order(out$loss) %in% 1:30)
print(selected_lines)
sweep(out$yHat[selected_lines,], 2, direction, '*')
```

**Example 4:**

```
## - - - - - - - - - - - - - - - - - - - - - - - - - - - - - - - - ##
##           Example 4
## - - - - - - - - - - - - - - - - - - - - - - - - - - - - - - - - ##
rm(list = ls())                                 # Clean R memory
setwd("your-path")
library(MPS)
library(ggplot2)
library(BGLR)

data(AdvEYT)

Y <- as.data.frame(Y[, c("YR_LUD", "GRNPRO", "GY_B5IR_F5IR_BEHT")])

X <- scale(X, center = TRUE, scale = TRUE)
Y2 <- Y
Y2$YR_LUD <- Y$YR_LUD*-1

# Model fitting using BGLR
ETA <- list(list(X = X, model = "BRR", saveEffects = TRUE))
model <- Multitrait(y = as.matrix(Y2), ETA = ETA, intercept = TRUE,
  resCov = list(type = "UN", saveEffects = TRUE), nIter = 100000, burnIn = 30000)

# Reading Posterior MCMC
B0 <- as.matrix(read.table(file = "mu.dat", header = FALSE))
B <- readBinMatMultitrait('ETA_1_beta.bin')
R <- as.matrix(read.table(file = "R.dat", header = FALSE))

# Evaluating Loss Function
t1 <- c(-30, -10, -5)   # 0.9, 0.75, 0.5
t2 <- c(13, 13.5, 14)
t3 <- c(101, 103, 104)
targets <- expand.grid(t1, t2, t3)
direction <- c(-1, 1, 1)
averages <- matrix(NA, ncol = 3, nrow = nrow(targets))   # estimated
averagesY2 <- matrix(NA, ncol = 3, nrow = nrow(targets)) # real

for(i in 1:nrow(targets)){

  out <- FastMPS(
    Xcand = X,
    B0 = B0,
    B = B,
    R = R,
    method = "kl",
    target = as.numeric(targets[i, ]),
    direction = direction)

    IDSel <- out$ranking %in% 1:20
    proms <- colMeans(out$yHat[IDSel,])
    promsY2 <- colMeans(Y2[IDSel,], na.rm = TRUE)
    averages[i,] <- proms
    averagesY2[i,] <- promsY2

}
```

**Example 5:**

```
## - - - - - - - - - - - - - - - - - - - - - - - - - - - - - - - - ##
##           Example 5
## - - - - - - - - - - - - - - - - - - - - - - - - - - - - - - - - ##

rm(list = ls())                                   # Clean Work space
setwd("your-path")                                # Working directory
library(MPS); library(BGLR)                       # Needed libraries
set.seed(752); data(AdvEYT)                       # Reproducibility and Load dataset
Y <- Y[,c('GY_BLHT', 'TKW', 'GRNPRO', 'SR_NJ')]   # Traits of interest
n <- nrow(Y)                                      # Number of candidates

# Allocating randomly missing phenotypic data in each trait
Y[sort(sample(1:n, size = 70, replace = FALSE)), 1] <- NA
Y[sort(sample(1:n, size = 50, replace = FALSE)), 2] <- NA
Y[sort(sample(1:n, size = 60, replace = FALSE)), 3] <- NA
Y[sort(sample(1:n, size = 20, replace = FALSE)), 4] <- NA
Y2 <- Y                   # Copy of phenotypic records
Y2[,4] <- Y2[,4] * -1     # Change sign in SR_NJ trait

# Center and scaling genomic info
X <- scale(X, center = TRUE, scale = TRUE)

# Specification of the linear predictor for BGLR
ETA <- list(list(X = X, model = "SpikeSlab", saveEffects = TRUE))

# Run the model
model <- Multitrait(y = Y2, ETA = ETA, intercept = TRUE,
 resCov = list(type = "UN", saveEffects = TRUE), nIter = 100000, burnIn = 30000)


# Reading Posterior MCMC of model parameters & direction
B0 <- as.matrix(read.table(file = "mu.dat", header = FALSE))# Overall mean
B <- readBinMatMultitrait('ETA_1_beta.bin')                 # Regression coefficients
R <- as.matrix(read.table(file = "R.dat", header = FALSE))  # Residual cov. matrix
direction <- c(1,1,1,-1)                                   # Direction of improvement
out <- FastMPS(Xcand = X, B0 = B0, B = B, R = R,       # Evaluation of Loss Function
               method = "kl", direction = direction)

idToSelect <- out$ranking %in% 1:40            # ID of selected
yHat <- out$yHat                               # Predicted BVs
yHat[,4] <- -1*yHat[,4]                        # Reverse order of BVs for trait 4

colMeans(yHat[idToSelect,])                    # Average BVs of selected

colMeans(yHat[!idToSelect,])                   # Average BVs of non-selected

NAsBySelLines <- apply(Y2[which(idToSelect),], 1, function(x) sum(is.na(x)))
IDSel <- which(idToSelect)
print(paste0(IDSel, '(', NAsBySelLines, ')'))
```

**Example 6:**

```
## - - - - - - - - - - - - - - - - - - - - - - - - - - - - - - - - ##
##           Example 6
## - - - - - - - - - - - - - - - - - - - - - - - - - - - - - - - - ##
rm(list = ls())                                        # Clean Work space
setwd("~/Desktop/R Package/Ejemplos Finales/example6") # Working directory
library(MPS); library(BGLR); library(tidyverse)        # Needed libraries
load('MultiEnvWheat.RData')                            # Load dataset
df <- Trigo.Data[,c(1,2,3)]                            # Keep only DTHD trait

PhenoEnv <- as.data.frame(pivot_wider(df, names_from = Env, values_from = DTHD))

# Seed for reproducibility
set.seed(647)

# Geno and Pheno data
G <- as.matrix(G)

# Given records were adjusted by experimental design there are
# negative values, here we sum 100 units to put in positive values
Y <- PhenoEnv[,2:4] + 100
rownames(Y) <- PhenoEnv$Gid

# Simulating some NAs entries that correspond to candidates
idNA <- sample(x = 1:nrow(Y), size = nrow(Y)*0.2)
YNA <- Y
YNA[idNA, ] <- NA

# Design matrix for individuals. It connects individuals with environments
GID <- factor(rep(rownames(Y), ncol(Y)), levels=rownames(Y))
Zg <- model.matrix(~GID-1)

# Design matrix for environments. Used in the multi-environment R-Norm model
envID <- factor(rep(colnames(Y),each = nrow(Y)), levels = colnames(Y))
ZE <- model.matrix(~envID-1)

#  Covariance structure for effects
ZgGZgt <- Zg %*% G %*% t(Zg)    # Genetic effect
ZEZEt <- tcrossprod(ZE)         # Environmental effect

# Eigen decomposition (to speed computational time)
eigen_G <- eigen(G)
eigen_G0 <- eigen(ZgGZgt)

# Interaction terms (MxE model)
MxE_eigen <- vector("list",ncol(Y))
for(env in 1:ncol(Y)){
    tmp <- rep(0,ncol(Y)) ; tmp[env] <- 1; G1 <- kronecker(diag(tmp),G)
    MxE_eigen[[env]] <- eigen(G1)
}

n <- nrow(YNA)
nEnv <- ncol(YNA)
y <- as.numeric(unlist(YNA))

# Number of iterations and burn-in for Bayesian models
nIter <- 30000; burnIn <- 2000

# MxE interaction model
ETA <- list(list(~envID-1,model="FIXED"))
ETA[[2]] <- list(V=eigen_G0$vectors,d=eigen_G0$values,model='RKHS')

# Adding interaction terms
for(env in 1:nEnv){
    eigen_G1 <- MxE_eigen[[env]]
    ETA[[(env+2)]] <- list(V=eigen_G1$vectors,d=eigen_G1$values,model='RKHS')
}

# Model Fitting
fm <- BGLR(y=y,ETA=ETA,nIter=nIter,burnIn=burnIn)

# Retrieving point estimates
B0 = fm$mu + fm$ETA[[1]]$b
yHat = matrix(fm$yHat,ncol=nEnv)[idNA, ]
v1 = fm$varE + fm$ETA[[3]]$varU
v2 = fm$varE + fm$ETA[[4]]$varU
v3 = fm$varE + fm$ETA[[5]]$varU
R = diag(c(v1, v2, v3))

# Eval loss function
out <- ApproxMPS(B0 = B0,
                 yHat = yHat,
                 R = R,
                 method = "kl")


# Only 40% of candidates is selected
idSel = out$ranking %in% 1:20

meansSel = colMeans(out$yHat[idSel,])
meansNoSel = colMeans(out$yHat[!idSel,])
dif1 = (meansSel - meansNoSel)
dif2 = (meansSel -  B0)
dif3 = (meansSel -  fm$mu )

dfToPlot = matrix(c(dif2, dif3), nrow = 2, byrow = TRUE)
colnames(dfToPlot) = colnames(Y)
rownames(dfToPlot) = c('With respect overall mean', 'With respect environment means')

barplot(dfToPlot,
        main = "",
        xlab = "Environments",
        ylab = "Difference in Average BVs",
        beside = TRUE,
        legend.text = rownames(dfToPlot),
        args.legend = list(bty = "n", x = "topleft", ncol = 1))
```
